# Health-service performance of TB treatment for indigenous and non-indigenous populations in Brazil: a cross-sectional study

**DOI:** 10.1186/1472-6963-14-237

**Published:** 2014-05-23

**Authors:** Everton Ferreira Lemos, Aline Mara da Silva Alves, Giovana de Castro Oliveira, Marcella Paranhos Rodrigues, Natália Daiane Garoni Martins, Julio Croda

**Affiliations:** 1 Faculdade de Ciências da Saúde, Universidade Federal da Grande Dourados, Rodovia Dourados – Itaúm. Km 12, Dourados, Mato Grosso do Sul 79804-970, Brazil

**Keywords:** Tuberculosis, Indigenous, Prevention, Control, Health services

## Abstract

**Background:**

Health-service evaluation studies are fundamental for proposing interventions and ensuring improvements in healthcare quality. The present study assesses the performance of health services for indigenous and non-indigenous populations with regard to tuberculosis (TB) control.

**Methods:**

Interviews with TB patients who underwent treatment between 2009 and 2011 were conducted using the *Primary Care Assessment Tool* adapted for TB care in Brazil.

**Results:**

Primary healthcare (PHC) was the first treatment for most patients at symptom onset, and the diagnoses were typically performed by specialized services. Many patients experienced delayed TB diagnoses that required more than three medical appointments (51% and 47% for indigenous and non-indigenous populations, respectively). Indigenous people received social support, such as basic-needs grocery packages (2.19 ± 1.63 vs. 1.13 ± 0.49 for non-indigenous people, p < 0.01) and home visits from health professionals, with an emphasis on the performance of directly observed treatment strategies (DOT; 4.57 ± 0.89 vs. 1.68 ± 1.04 for non-indigenous people, p < 0.01).

**Conclusions:**

Regardless of the differences between indigenous and non-indigenous populations, the time needed to receive a TB diagnosis was unsatisfactory for both groups. Furthermore, DOT must be performed with better coverage among non-indigenous patients.

## Background

Tuberculosis (TB) is a worldwide health problem. One-third of the world’s population may be infected with *Mycobacterium tuberculosis.* Brazil is among the 22 countries with the highest incidence of TB, with a rate of 43 cases per 100,000 inhabitants in 2010 [1]. In the world and in Latin America, studies show a high incidence of TB in indigenous populations, exceeding that of the general population [2-7].

Since 1998, TB control has been the responsibility of Brazil’s primary healthcare [8]. The decentralization strategy aims to offer health services related to disease diagnosis and to institute the DOT for all TB patients [9,10]. In recent years, many efforts have been made to increase the coverage of Primary Health Care (PHC). Although 50% of Brazilians have coverage for primary care [11], the majority of diagnoses of tuberculosis are performed in the emergency department or in secondary and tertiary hospitals [12,13]. PHC presents the longest time to diagnosis and the lowest proportion of diagnoses [14]. In addition, increased coverage does not result in an improvement in the success rate of treatment [15].

Operational studies are important because they provide information that may help services focus their practice more precisely and efficiently [16]. Although several studies have evaluated health services for tuberculosis control in Brazil [12-14,17-19], no study has evaluated the decentralization of healthcare in the context of the indigenous population.

Dourados was a priority city for disease control determined by the National TB Control Program and includes the largest indigenous population of the State of Mato Grosso do Sul with a high incidence of TB. The incidence of TB in the urban population of Dourados is 44 cases per 100,000 inhabitants, and the incidence among the indigenous people of this municipality is even higher, at 230 per 100,000 inhabitants [20].

Before 2000, TB treatment for indigenous patients was hospitalization for several months, and the default rate was 20% [21,22]. Croda et al. [20] retrospectively evaluated the performance of health services for this population and found that with the implementation of the Directly Observed Treatment Strategy (DOTS), there was a considerable reduction in default to treatment of 2%. However, this study has some limitations and potential confounding factors. The secondary database is also used for epidemiological surveillance; therefore, the results found in this study are limited by the absence of important variables related to treatment default, as described in the literature, such as social characteristics, smoking, malnutrition, housing conditions, intravenous drug use, socioeconomic status, and access to health services. Another relevant limitation is the incompleteness of some of the information, such as the number of medical appointments and the time required to establish the diagnosis of tuberculosis.

To clarify these initial findings, a cross-sectional study was proposed to evaluate the performance of two different TB control programs (indigenous and non-indigenous) in Dourados using a standardized tool to evaluate the performance of health services.

## Methods

### Study design and sample

**Figure 1:**
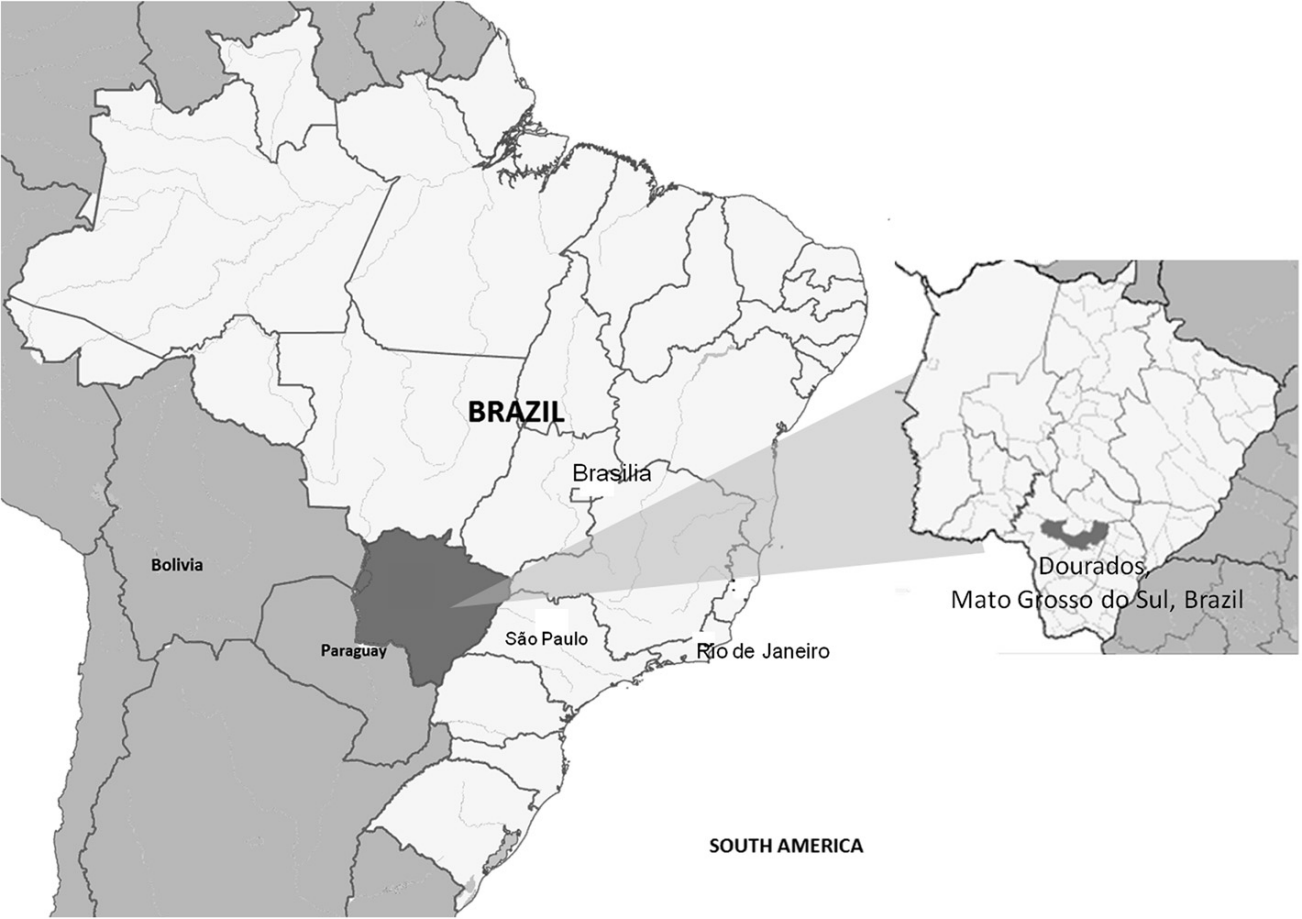
Map of Localization of Dourados, Brazil.

This cross-sectional study was conducted in Dourados, Mato Grosso do Sul (MS). This city has 196,035 inhabitants, including 12,602 indigenous people (Guarani-Kaiowá ethnicity) who reside in the indigenous reserves of Bororó and Jaguapiru, the second largest indigenous population of the country.Dourados serves as a reference for 34 cities in the South region of the state in terms of healthcare, agriculture, and trading (Figure 1).

Regarding the organization of care for TB, two models of care are identified at the study site. The Municipal Secretary of Health cares for the non-indigenous population, including primary care centers, specialized centers, and University Hospital. For the Indians, the Special Indigenous Health Agency (SESAI) is responsible for the management of a health and tuberculosis control program that manages the diagnosis and treatment of TB and notifies the Information System for Notifiable Diseases (SINAN) about TB infections in this population.

The diagnosis of TB is accomplished by smear, culture, and X-rays [23]. The BHU offers pots for indigenous people to collect sputum and directs patients to the Mission Hospital, which is equipped to provide X-rays and bacteriological examinations. Smear sputum microscopy of non-indigenous patients is performed by the laboratory of the municipal Department of Health, and patients are referred to specialized centers and University Hospital for X-rays.

The sample size required for the T-test was 49 patients, calculated using Statistica software (using the commands two means, t-Test, Ind. Samples); mu1 = 2; mu = 3; sigma = 1.5; probability of type I error = 0.05; and power 0.9.

The inclusion criteria were diagnosis with TB by the Notifiable Diseases Information System (Sistema de Informação de Agravos e Notificação; SINAN) and residence in Dourados during treatment between June 1, 2009 and July 31, 2011. Prisoners and patients with diagnosis changes were excluded from this study. Study attrition was due to patients who chose not to participate and those who died or abandoned treatment prior to the interview.

Medical students received specific training, including a detailed description of the study and examples of interviewing techniques. They collected data during two periods: in the first month for the collection of sociodemographic and economic data and in the sixth month for the evaluation of health services data. Both data sets were collected at the patients’ homes after obtaining informed consent and explaining the purpose of the study.

Indigenous health workers were present at all of the interviews conducted in the indigenous communities. These employees helped search for households, introduced the study staff, and translated when necessary.

### Data collection tool

The instrument includes questions to elicit sociodemographic, clinical, and epidemiologic information, the location and type of treatment, and specific questions for each PHC component. The Primary Care Assessment Tool (PCAT) [24] was adapted, validated, and transferred by Villa and Ruffino Neto (2009) [25]. The PCAT is the only instrument that has been translated and validated to assess health services for TB control in Brazil. Therefore, it was chosen despite cultural barriers related to the indigenous population. The PCAT is divided into three sections.

I. “Access”, which is divided into the categories “access to diagnosis” and “access to treatment”, includes the locations of the basic health units (BHUs), the hours/days that they are open, their degree of tolerance for unscheduled consultations, and the extent to which the population perceives that the access is convenient [26].

II. “Bond” measures the existence of a regular source of care and use over time [26].

III. “List of services” assesses arrangements for the patient to receive any type of required healthcare service [26].

The respondent (or legal representative) answered each question on the form using a five-point Likert scale.

### Data analysis

The variables were entered twice into the Epi-Data program database, Version 3.0 and were analyzed using the Statistical Analysis Software Version 9.1 (SAS Institute Inc., Cary, NC, USA). The double entry of the questionnaire identified mistakes in typing.

Frequency and mean distributions were used to describe the patients’ sociodemographic and clinical profiles. Means and standard deviations of the variables described using the Likert scale were analyzed and compared using the nonparametric Kruskal-Wallis test. These averages were evaluated on an intensity scale from one to five: (1) never, (2) almost never, (3) sometimes, (4) almost always, and (5) always. This order was reversed if necessary according to the observed variable. To evaluate the item “home visit”, the average number of times that health professionals delivered the drug was used, and the following scale values were assigned: (1) self-administered, (2) every 15 to 30 days, (3) 1 to 2 times a week, (4) 3 to 4 times a week, and (5) every day. All inquiries were made to all individuals. The no-answer condition was assigned a value of zero, “do not know”, which was not counted in the analysis of each question.

Despite the use of the Kruskal-Wallis test when comparing the differences between the Likert scales, the results were deliberately presented in means and standard deviations to facilitate the visualization of the data. Dichotomous and categorical data were analyzed using the chi-square or Fisher’s exact tests.

The PCAT’s reliability was assessed using its internal consistency and verified by Cronbach’s alpha. Values between 0.70 and 0.90 were considered acceptable [27].

### Ethical considerations

The Ethics Committee of the Federal University of Grande Dourados (nº 002/2009) and the National Committee for Research Ethics/National Health Committee (Comissão Nacional de Ética em Pesquisa/Conselho Nacional de Saúde - CONEP/CNS 379 627/09) approved the present study.

## Results

**Figure 2:**
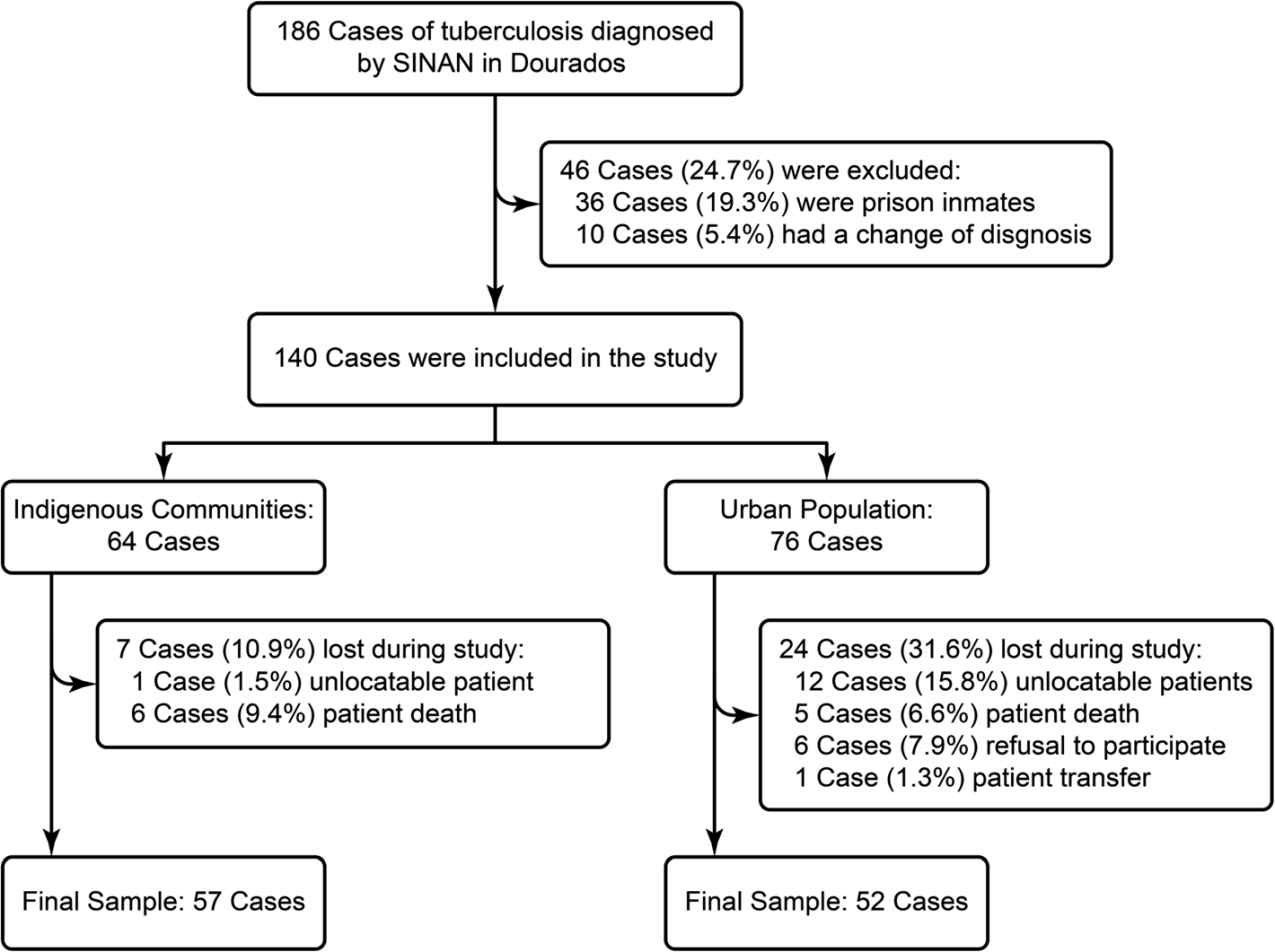
The recruitment of tuberculosis cases.

One hundred and eighty-six patients with TB were notified during the study period, and 46 patients (24.7%) were excluded for not having a confirmed diagnosis or being in a seclusion regimen. Among the remaining 140 patients, 13 had follow-up losses due to our inability to locate, 11 died before the end of treatment, and six patients refused to participate in the study. Four non-indigenous patients presented treatment default. The cure rate was 90.6% *versus* 81.5%, mortality rate 9.3% versus 6.7% and default rate of 0% versus 5.2% for indigenous and urban populations, respectively. Thus, 109 patients constituted the sample evaluation of health services (Figure 2).

Regarding losses, no significant differences were found between the two groups. A total of 12 children required legal representatives to answer the PCAT, including 2/52 (3.8%) in the non-indigenous population and 10/57 (17.5%) in the indigenous population. There was no difference between those who self-responded and those who did not. The internal consistency coefficient (Cronbach’s alpha) ranged from 0.71 to 0.80 for the Likert scale variables.

Among the 109 patients with TB who were interviewed, men constituted the majority (60%). The indigenous patients had a lower mean age, less education, lower social class, and lower income per capita as well as more individuals per room compared with non-indigenous patients. Compared with the indigenous patients, the non-indigenous patients had higher rates of alcoholism, drug abuse, and human immunodeficiency virus/acquired immunodeficiency syndrome (HIV/AIDS) (Table 1).

**Table 1:** Sociodemographic, economic, clinical, and epidemiologic characteristics of indigenous and non-indigenous patients with TB in Dourados, Mato Grosso do Sul (MS), Brazil between June 2009 and July 2011 (N = 109)

**Sociodemographic, economic, epidemiologic and clinical variables**	**Indigenous**	**Non-indigenous**	**p-value**
	**n = 57**	**n = 52**	
Sex male	31 (55%)	34 (66%)	0.07
Age, mean ± SD	30.29 ± 25.60	42.02 ± 37.58	**<0.01**^**1**^
Have a partner	30 (53%)	21 (40%)	0.05
No education	42 (74%)	14 (27%)	**<0.01**
Monthly income per capita (U.S. dollars), mean ± SD	55.82 ± 61.67	226.8 ± 194.1	**<0.01**^**1**^
Social class (ABEP)^2^, mean ± SD	5.03 ± 4.14 (E)	14.06 ± 5.26 (C2)	**<0.01**^**1**^
Works at the sugar cane factory	9 (16%)	0 (0%)	**<0.01**
Knows someone with TB	30 (53%)	14 (27%)	**<0.01**
Agglomeration^3^, mean ± SD	2.23 ± 1.69	0.99 ± 1.67	**<0.01**^**1**^
Alcoholism	4/53 (8%)	10 (19%)	**0.03**
Smoking	49 (86%)	37 (71%)	**0.01**
Drug abuse	4 (7%)	11 (21%)	0.04
HIV positive	0 (0%)	6 (12%)	0.06
Pulmonary form of TB	51 (90%)	44 (85%)	0.14
Diagnostic			
Smear positive	48 (84%)	40/48 (83%)	0.98
Smear negative and culture positive	4/56 (7%)	2/48 (4%)	0.46

^1^Kruskal-Wallis Test.

^2^ABEP: Brazilian Association of Research Companies.

^3^Agglomeration: persons per room.

Patients with TB in Dourados most often sought PHC at symptom onset (75% of indigenous patients and 65% of non-indigenous patients), and their diagnoses were primarily performed at specialized services (60% and 65% for indigenous and non-indigenous patients, respectively). A delayed diagnosis of TB, which required more than three medical appointments (46% for indigenous patients and 44% for non-indigenous patients) and over five weeks (51% for indigenous patients and 47% for non-indigenous patients), was found with regard to both PHC and specialized services. For indigenous people, home visits by health professionals were offered more frequently, highlighting the performance of the strategies of directly observed treatment (DOT) (Table 2).

**Table 2:** Access to diagnosis and treatment for patients in Dourados from June 2009 to July 2011 (N = 109)

**Access to diagnosis**		**Indigenous**		**Non-indigenous**		**p-value**
		**n**		**n**		
Initially sought PHC	**(%)**	57	43 (75)	48	31 (65)	0.32
Diagnosis in specialized service	**(%)**	57	34 (60)	48	31 (65)	0.75
Diagnosis time ≥ 5 weeks	**(%)**	53	27 (51)	47	22 (47)	0.69
Number of appointments before diagnosis ≥ 3 times	**(%)**	50	22 (46)	48	22 (44)	0.85
Sought health services closer to home?	**Mean ± SD**	57	1.42 ± 1.48	50	2.26 ± 1.71	<0.01*
Waited more than 1 hour to be seen?	**Mean ± SD**	57	2.89 ± 1.99	50	2.40 ± 1.69	0,22*
Had difficulties reaching health services?	**Mean ± SD**	56	3.75 ± 2.14	50	3.70 ± 1.77	0,94*
Spent money on transportation to reach health services?	**Mean ± SD**	57	4.40 ± 1.79	51	3.80 ± 1.76	<0.01*
Missed work or appointments for the medical appointment?	**Mean ± SD**	57	2.82 ± 2.19	51	3.39 ± 1.83	0,10*
**Access to treatment**						
Ability to obtain an appointment in 1 day	**Mean ± SD**	57	3.70 ± 1.96	52	3.82 ± 2.18	0,72*
Underwent TB treatment at the health facility closest to home?	**Mean ± SD**	57	4.28 ± 1.71	52	4.00 ± 1.65	0,11*
How often does a health professional travel to your home to deliver TB medication?^†^	**Mean ± SD**	56	4.57 ± 0.89	51	1.68 ± 1.04	<0.01*

Access to diagnosis. 1 = always, 2 = almost always, 3 = sometimes, 4 = rarely, and 5 = never. Access to treatment. 1 = never, 2 = almost never, 3 = sometimes, 4 = almost always, and 5 = always.

^†^1 = Self-administered, 2 = every 15 to 30 days, 3 = 1 to 2 times a week, 4 = 3 to 4 times a week, and 5 = everyday.

*Kruskal-Wallis Test.

Regarding compliance/bond, the indigenous patients with TB felt that they were not as well understood as non-indigenous patients when asking questions (Table 3).

**Table 3:** Compliance/bond for TB patients in Dourados from June 2009 to July 2011(N = 109)

**Compliance/bond**		**Indigenous**		**Non-indigenous**		**p-value**
		**n**		**n**		
When going to the clinic for a TB consultation, do the same professionals see you?	**Mean ± SD**	56	4.75 ± 0.82	51	4.65 ± 0.88	0.21*
If you have questions about your treatment, are you able to speak with the same professionals who treat you at the clinic?	**Mean ± SD**	57	4.63 ± 1.24	51	4.38 ± 1.39	0.26*
After asking a question, do you understand the answer?	**Mean ± SD**	57	4.33 ± 1.27	51	4.70 ± 1.20	0.04*
Do the health professionals at the clinic answer your questions clearly?	**Mean ± SD**	57	4.36 ± 1.35	51	4.76 ± 1.06	0.15*
Do the health professionals at the clinic provide enough time to address your questions or concerns?	**Mean ± SD**	57	3.98 ± 1.51	50	4.68 ± 1.13	0.01*
Do the health professionals address your other health problems during the appointment?	**Mean ± SD**	57	2.50 ± 1.65	51	2.82 ± 1.92	0.51*
Do the health professionals discuss the TB medication?	**Mean ± SD**	57	4.43 ± 1.29	51	4.23 ± 1.49	0.53*
Do the health professionals ask about all medications that you are taking?	**Mean ± SD**	57	3.31 ± 2.04	51	3.19 ± 1.89	0.85*
What is your opinion of the healthcare team that treats you? Rate them from 1 to 5.	**Mean ± SD**	53	4.37 ± 1.16	50	4.50 ± 1.11	0.47*
Do you feel that the healthcare team has any kind of prejudice?	**(%)**	56	(21)	47	(23)	0.81

Compliance/bond. 1 = never, 2 = almost never, 3 = sometimes, 4 = almost always, and 5 = always.

*Kruskal-Wallis Test.

Fewer indigenous patients had access to HIV/AIDS testing compared with non-indigenous patients. Furthermore, the indigenous patients were less likely to spend money to reach the BHUs, and they were treated at the BHU closest to their homes. Moreover, the indigenous patients were more likely to receive social support, such as basic-needs grocery packages and home visits from health professionals, when the performance of DOTS was emphasized compared with non-indigenous patients (Table 4).

**Table 4:** List of services for patients in Dourados from June 2009 to July 2011 (N = 109)

**List of services**		**Indigenous**		**Non-indigenous**		**p-value**
		**n**		**n**		
Does the medical team that accompanies you during TB treatment…	**(%)**	53	20 (38%)	45	37 (82%)	**<0.01**
Offer you HIV/AIDS testing?						
Offer a flask for sputum examination each month for control of your TB?	**Mean ± SD**	57	3.85 ± 1.59	51	3.88 ± 1.70	0.76*
Offer monthly consultations for TB treatment and control?	**Mean ± SD**	55	3.85 ± 1.55	51	3.90 ± 1.59	0.84*
Offer basic-needs grocery packages or food vouchers?	**Mean ± SD**	57	2.19 ± 1.63	51	1.13 ± 0.49	<0.01*
Offer transportation vouchers?	**Mean ± SD**	57	1.45 ± 1.51	51	1.11 ± 0.65	0.41*
Explain the symptoms of TB?	**Mean ± SD**	54	3.70 ± 1.75	51	3.54 ± 1.96	0.80*
Explain TB transmission?	**Mean ± SD**	56	3.39 ± 1.73	51	3.76 ± 1.89	0.26*
Explain TB treatment?	**Mean ± SD**	56	4.53 ± 1.06	50	4.26 ± 1.38	0.35*
Explain your other health problems?	**Mean ± SD**	56	2.42 ± 1.80	51	2.33 ± 1.95	0.46
Conduct home visits for reasons other than TB?	**Mean ± SD**	56	3.05 ± 1.77	50	2.08 ± 1.53	<0.01*
Are the health professionals at the clinic always available when you need them?	**Mean ± SD**	57	4.68 ± 1.02	51	4.66 ± 1.69	0.72*
Are there groups of patients who discuss TB at the clinic where you are being treated?	**Mean ± SD**	56	4.62 ± 3.75	51	4.80 ± 4.00	0.83*

List of services. 1 = never, 2 = almost never, 3 = sometimes, 4 = almost always, and 5 = always.

*Kruskal-Wallis Test.

## Discussion

Despite the differences between the indigenous and non-indigenous groups, the time to diagnosis was shown to be unsatisfactory in both groups. Previous studies have also found delayed TB diagnoses [28-32]. As a result of this delay, the risk of transmission and the possibility of death remain high [13]. The evolution to cure was higher among the indigenous populations (90.6 vs 81.5%, the mortality rate was similar among indigenous and urban population (9.3% vs 6.7%). The default rate is 0% in the indigenous versus 5.2% in the urban populations. This findings suggesting the superiority of indigenous tuberculosis control program related to DOT covered [10].

Indigenous families are large and have more people per room than non-indigenous families (2.23 ± 1.69 vs. 0.99 ± 1.67; < 0.01). The size and number of rooms in the home together with aglomeration are factors that influence the risk of TB transmission [33] and can be associated with the high incidence of the diseases in the indigenous populations [20]. The risk of developing TB in households with four or more people is approximately three times higher compared to households with two people or fewer [33]. Recent findings by Welch and Coimbra Jr. [34], analyzing cultural perspectives of tuberculosis transmission and treatment among the Xavante of Mato Grosso State, Brazil support this idea.

In this study, home visits for the treatment of tuberculosis occurred less frequently in the non-indigenous population (4.57 ± 0.89 vs. 1.68 ± 4.1 for non-indigenous people, p <0.01). A study conducted by Croda et al. [20] observed lower rates of DOT coverage for this population when compared with the indigenous population (non-indigenous 35% vs. indigenous 92%, p <0.001), further highlighting the higher default rates (17% vs. 2%; p <0.001). Amaral et al. [35] evaluated the decentralization program to control tuberculosis between 2003 and 2006 and found a worsening in the percentage of default (7.7% to 10.2%) and a significant increase in the percentage of deaths (3.0% to 15.4%). Despite decentralization, primary care does not make frequent home visits, which hinders compliance and the completion of treatment.

The WHO highlights the importance of the organizational and performance dimensions of healthcare services (HS), stating that the problem does not lie in detecting or treating TB, but in how healthcare services are organized to detect and treat cases of TB [36]*.* This perspective emphasizes the need to expand the diagnosis of tuberculosis and to offering more X-rays and smears in primary healthcare. This approach would provide the opportunity for earlier diagnosis by decreasing the time to diagnosis and the number of late diagnoses in emergency and secondary or tertiary services.

Although the majority of patients sought medical care at the PHC, the current study showed that most TB diagnoses were performed in emergency departments and hospitals. The healthcare model found in the municipality explains this trend because the Mission Hospital specializes in TB diagnosis among the indigenous population, whereas University Hospital primarily serves the non-indigenous population. These findings are similar to several studies conducted in Brazil [12,13].

Supplying HIV/AIDS exams did not result in satisfactory performance, particularly among the indigenous population. In Brazil, it is recommended that the investigation of HIV should be supplied to 100% of TB patients [10]. This exam is performed only in the Sexually Transmitted Diseases reference service, with no corresponding service in indigenous reserves. The implementation of rapid HIV testing in primary care service would facilitate access to the exam, as observed in other studies with neglected populations [37-39].

The indigenous patients felt that they were not as well understood as the non-indigenous patients when they asked health questions. Indigenous people consider diseases to be individual, a sign of social or world disorder, which contradicts the understanding of occidental biomedicine [40]. Sensitivity to the culture and its conceptions about the health-disease process is essential for improving the doctor-patient relationship [41,42].

This study has limitations related to possible sample selection bias because of participant attrition related to death and treatment default, which led to the non-completion of the questionnaire and the exclusion of data from the analyses. Communication difficulties with the indigenous community were not reduced even in the presence of an indigenous health worker. This difficulty was due to both the low level of education and the language barriers, which resulted in a lack of understanding of certain terms and phrases as well as difficulty in responding according to the Likert scale. The instrument is extensive and requires response measures. The application of the PCAT for the indigenous population may not be the best alternative, although it is the only validated instrument in Brazil for the evaluation of health services in primary tuberculosis.

Despite these limitations, this study showed that after operating the decentralization process, there were improvements in TB control among the indigenous population, with home visits providing a reduction in the incidence rate. In the non-indigenous population, despite decentralization, there is a lack of access to diagnosis and a need for greater coverage and commitment of health professionals in providing the DOT.

## Conclusion

Despite the differences between the indigenous and non-indigenous groups, the time to diagnosis was shown to be unsatisfactory in both groups. In particular, the rapid HIV-AIDS test should be offered through primary healthcare, especially in indigenous communities. For non-indigenous people, improvements in access to diagnosis and treatment are necessary, especially with regard to greater coverage of DOT. Incentives for the strengthening of primary healthcare with the co-responsibility of other services may contribute to effective improvements in TB treatment.

## Abbreviations

PHC: Primary healthcare; DOTS: Directly Observed Treatment Strategy; UH: University Hospital; PCAT: Primary care assessment tool; SESAI: Special Indigenous Health Agency (Secretaria de Saúde Indígena); SINAN: Notifiable diseases information system (Sistema de Informação de Agravos e Notificação); TB: Tuberculosis; BHU: Basic health unit; HS: Healthcare services.

## Competing interest

The authors declare that they have no competing interests.

## Authors’ contributions

EFL coordinated and drafted the manuscript, designed the study, and participated in the data analysis and discussion. JC coordinated the research project, designed the study, analyzed the data, and contributed to the discussion. AMSA, GCO, MPR, and NDGM collected the data, analyzed the data, and contributed to the discussion. All authors interpreted the results and read and approved the final manuscript.

## Pre-publication history

The pre-publication history for this paper can be accessed here:

http://www.biomedcentral.com/1472-6963/14/237/prepub
